# The structure of IL2 bound to the three chains of the IL2 receptor and how signaling occurs

**DOI:** 10.1186/1476-9433-5-3

**Published:** 2006-08-14

**Authors:** Kendall A Smith

**Affiliations:** 1 The Division of Immunology, Department of Medicine, Weill Medical College, Cornell University, New York, NY, 10021, USA

## Abstract

The interleukin-2 molecule and receptor were the first of the interleukins to be discovered and characterized at the molecular level. Now after 20 years of effort, two groups have succeeded in determining the structure of IL2 bound to the external domains of the three receptor chains in a quaternary complex. What do we know now that we did not know before this structural information was available, and how do these new data help us to develop new therapies?

In the early 1980s, soon after we had characterized the IL2 molecule as a 15.5 kDa variably glycosylated protein[1,2], had purified it to homogeneity[2], and had discovered and characterized the IL2 receptor (IL2R)[3], our attention turned to trying to determine exactly how IL2 binding to its receptor leads to signals that promote cellular proliferation. One approach to this question involved determining the 3-dimensional (3D) structures of the IL2 molecule and the IL2R via X-ray crystallography. However, it took more than a decade for us and others to discover that the IL2R is comprised of three distinct non-covalently linked chains, termed alpha (α, CD25)[4], beta (β, CD122) [5-7], and gamma (γ, CD132)[8]. Subsequently, as the cDNAs encoding each chain became available, we began to collaborate with Ian Wilson of the Scripps Research Institute to try to determine the structures of these molecules. Instrumental in these experiments was Tom Ciardelli at Dartmouth, who constructed expression systems to produce large amounts of the proteins to be used in obtaining crystals of the molecules, which he also used in rigorous reduction experiments with binding studies using isolated soluble receptor molecules.

Anything is possible in science, but some things take a very long time. Indeed, crystals of IL2 bound to the IL2R α chain were readily achieved as early as 1989[9], but they remained recalcitrant to structure solution for many years. Moreover, the fact that the IL2R is comprised of three separate chains made the task of crystallizing all four proteins bound together exceedingly difficult. Twenty years after our experiments were initiated, Chris Garcia at Stanford with his team of two talented post docs, Xinquan Wang and Mathias Rickert, succeeded in determining the structure of IL2 bound to the external domains of the three IL2R chains in a quaternary complex[10]. Chris is a former postdoc of Ian Wilson, and he generously shared their refined coordinates with Ian, so that two other talented members of Ian's group, postdoc Deborah Stauber and graduate student Erik Debler, could finish the structure determination of the IL2/IL2R quaternary complex, which they also had also assembled and crystallized[11].

Before examining the new data, it is useful to summarize the information that we have gained over the past 25 years as to how the IL2/IL2R ligand/receptor complex functions. IL2 itself is a small globular glycoprotein comprised of four antiparallel α helices[12]. IL2 was the first cytokine found to mediate its effects via a cell surface binding site that satisfied all of the requirements to be termed a classic hormone receptor, originally defined by Langely in 1878 and 1905[13,14]. Thus, IL2 binds to the IL2R with high affinity, stereospecificity, and saturability. In other words, there are a finite number of sites expressed on activated lymphocytes that are capable of binding only IL2, among all of the other cytokines. In addition, IL2 signals the cell at the same low concentrations that lead to binding to the IL2R at steady state, thus satisfying the requirement that true receptors must signal a physiological response after binding the ligand at physiological concentrations. The question before us is how these classic receptor characteristics are created at the molecular level.

The high affinity of the IL2R (K_d _= 10^-11 ^M) results from a rapid association rate contributed by the α-chain (k_on _= 10^7 ^M^-1^sec^-1^), combined with a relatively slow dissociation rate (k_off _= 10^-4 ^sec^-1^) contributed by both the β and γ chains[15,16]. Accordingly, from this information, it was concluded that distinct areas of the IL2 molecule bind to each of the three receptor chains. Moreover, it was also found that the α chain does not participate in signaling, whereas both the β and γ chains are necessary for signaling[17,18]. One of the perplexing aspects of the SAR of IL2/IL2R binding was the contribution of the γ chain, in that it was discovered that the γ chain is a component of several other cytokine receptors, including the IL4R, IL7R, IL9R, IL15R, and the IL21R [19-21]. Exactly how each of these different, although similar, cytokines could actually bind to the same γ chain remained an enigma.

A series of reports from the Ciardelli group at Dartmouth over the 1990s using isolated receptor chains and Surface Plasmon Resonance (SPR) dissected the complex relationships between IL2 and the three receptor chains [22-27]). Their data supported a model in which the α and β chains pre-associate on the cell surface to form a pseudo-high affinity site with a faster on-rate and a slower off-rate than either of the individual subunits. Thus, the efficiency of ligand capture is facilitated by the formation of this α/β chain heterodimer. Moreover, these data are consistent with the IL2-induction of α chain expression[28], which results in a 10–20-fold excess of α chain vs. β chain expression, thereby favoring the formation of an α/β heterodimer on the cell surface via the law of mass action.

In addition, their data indicated that signaling only occurs subsequent to the recruitment of the γ chain to the IL2/α/β trimeric complex[25,27]. Even though the γ chain is only weakly able to interact with IL2 by itself (K_d _> 700 μM), when recruited to join the heterotrimer of IL2 bound to the α/β heterodimer, the γ chain reduces the off-rate of the bound ligand substantially by forming a stable quaternary ligand/receptor complex. Thus, the model predicted that the mechanism controlling the duration of receptor signaling is the rate of ligand/receptor internalization (t_1/2 _= 15 minutes), rather than ligand dissociation (t_1/2 _= 45 minutes), as we had proposed originally[29]. This is important, given that the cell counts the total number of triggered IL2Rs, which is responsible for signaling a quantal (all-or-none) cellular response [30-32].

An additional view of how IL2 interacts with the three receptor chains was reported by Garcia's group using Isothermal Titration Calorimetry and Multi-Angle Light Scattering[33]. Even in the absence of IL2, they found low affinity binding of the α and β chains (K_d _= 278 nM), thereby supporting the Ciardelli SPR studies. Also, similar to Ciardelli's SPR results, there was no binding between the α and γ chains or the β and γ chains in the absence of IL2. Also, similar to previous IL2 binding studies, a definite affinity of IL2 for isolated α chains (K_d _= 10 nM) and isolated β chains (K_d _= 144 nM), but little or no affinity for IL2 binding to isolated γ chains was found using these thermodynamic techniques. These investigators interpreted their data as consistent with IL2 binding rapidly first to isolated α chains, followed by the α-bound IL2 being stabilized by binding to isolated β chains. Alternatively, their data were also consistent with IL2 binding to a preformed α/β dimeric complex as proposed by Ciardelli. All of the data were consistent with the IL2/α/β trimeric complex binding to the γ chain to form the final signaling complex.

The crystallization of IL2 bound to the external domains of the three receptor chains in a quaternary complex[10,11] revealed that the sites on IL2 that interact with the three chains of the IL2R do not overlap, except for a small but significant region, as predicted from the earlier binding and SPR studies. The 4-helix bundle of IL2 is clamped between the elbow regions of the β and γ chains. The IL2 molecule is held decisively between these two receptor chains, which converge to form a Y shape, with IL2 bound in the fork of the Y. In contrast, the other side of the IL2 molecule binds to the α chain, which Garcia's team had previously delineated from binary IL2/α chain crystals[34]. It is also noteworthy that the α chain itself does not contact either the β or γ chains in the crystal structure.

The crystal structure essentially does not help in discriminating whether IL2 binds first to the α chain alone or to a preformed α/β heterodimer. However, the pseudo-high affinity of the IL2/α/β trimeric complex (i.e. K_d _~300 pM) clearly indicates that the trimeric complex is more stable than either IL2 bound to the α chain alone (K_d _= 10 nM) or to the β chain alone (K_d _= 450 nM) as shown by Ciardelli's data. In any event, the IL2/α/β trimer would then recruit the γ chain into the quaternary complex capable of signaling, which is facilitated by the large composite binding site on the IL2-bound β chain for the γ chain. Since only a few residues of IL2 interact with both β and γ chains, binding of IL2 may induce conformational changes in the β chain that would further promote recruitment of the γ chain. This interpretation is consistent with data obtained by the Ciardelli group using SPR[27]. Moreover, the surface area of IL2-γ chain contact is the smallest of the three receptor chains (970 Å^2^), while the surface area of the β-γ chain contact is larger (1,640 Å^2^). Accordingly, the γ chain can serve as a receptor subunit for many similar but different cytokines by a cytokine-dependent binding of other *receptor *subunits to the γ chain, rather than binding of the cytokines themselves to the γ chain. Furthermore these data provide support for the search for inhibitors of the β chain-γ chain interaction as new immunosuppressants.

All of this structural information is entirely consistent with what we know about IL2R signaling, in that the cytoplasmic domain of the β chain is complexed with the JAK1 tyrosine kinase, while the γ chain cytoplasmic domain is complexed with the JAK3 kinase[18]. Thus, only when IL2 binding brings the external domains of these two receptor chains into close proximity, can signaling occur by trans phosphorylation of their cytoplasmic domains. In addition, as emphasized by Ciardelli's SPR data and by Garcia's energetics data, these structural data indicate that the quaternary ligand/receptor complex is very stable and signaling will continue until the receptor with bound ligand is internalized and degraded.

In the context of all of these new structural data, an earlier report from Marrack's group[35] is perplexing, in that it showed that the injection of mice with monoclonal antibodies (mAb) reactive with IL2 that block the IL2/IL2R α chain interaction, thereby inhibiting IL2-promoted T cell proliferation *in vitro*, actually ***increased ***the number of proliferating CD8+ T cells *in vivo*, instead of decreasing them as expected. These authors interpreted their results as showing that IL2 functioned to actually ***kill ***CD8+ T cells *in vivo*. Of course this interpretation is based upon the assumption that the anti-IL2 should have blocked IL2-α chain binding and T cell activation *in vivo*, as it did *in vitro*.

Now, a more recent report from Sprent's group[36] returned to this paradox; i.e. that the proliferation of CD8+ T cells with a memory phenotype (defined by a high expression of the IL2R β chain, but low or absent levels of the IL2R α chain), can be increased by injecting either IL2 itself or an IL2-reactive, inhibitory mAb. Since the interpretation by Marrack's group was counterintuitive, Sprent repeated their experiments, and also found an increase in CD8+ T cells using the same mAb. However, in addition, they used IL2 gene deleted mice, and found that the enhanced proliferative effect of the anti-IL2 treatment was abolished in this setting.

Consequently, they then hypothesized that perhaps the IL2-reactive mAb functioned to actually increase biological activity of endogenous IL2 via the formation of immune complexes with IL2, thereby preserving and promoting IL2 activity. This was in fact found to be the case, and the simultaneous administration of IL2 and IL2-reactive mAb resulted in a dramatic increase (> 100-fold) in the total numbers of CD8+ T cells, and NK cells as well, which also express both the β chain and the γ chain of the IL2R.

On the basis of these data and others, Sprent concluded that the stimulatory IL2-reactive mAb binds to a site on IL2 that occludes its binding to the α chain, but does not impair binding to the β chain. In other words, the IL2-reactive MoAb takes the place of the α chain on cells in vivo, and presents IL2 to the β and γ chains, thereby stimulating proliferation, particularly of memory CD8+ T cells that already express the β/γ chains as part of the IL15R. In light of the new structural data, Sprent's interpretation makes perfect sense. In addition, as Fab_2 _fragments of the IL2/mAb complex were inefficient in promoting *in vivo *T cell proliferation, these findings are consistent with IL2/mAb immune complexes on APCs substituting for the cell-bound α chain on T cells serving as a ligand carrier and/or capturer, as suggested by Stauber et. al. [11].

If further investigation supports these findings, the use of cytokine immune complexes as immune stimulants could very well markedly improve immunostimultatory therapy, in that the cytokine immune complexes have a much improved in vivo half-life compared with cytokines alone. For example, the half-life of IL2 administered intravenously is only ~10 minutes due to distribution into the total body extracellular space, which is large, ~15 L in an average sized adult. Subsequently, IL2 is metabolized by the kidneys with a half-time of ~2.5 hours. By comparison the in vivo half-life of administered Ig is measured in several days if not weeks[37].

Still left obscure regarding the function of the quaternary IL2/IL2R complex is the exact molecular rearrangements that occur in the intracytoplasmic domains of the IL2R chains, especially the β and γ chains, in that these domains have not yet been crystallized. Thus far, all efforts to date to crystallize these domains have been fruitless, and may indicate that the cytoplasmic tails do not adopt a permanent well-defined structure, which is a prerequisite for crystallization. However, two additional new reports on signaling from the intracytoplasmic domains via the JAKS to STAT5a/b have recently underscored the importance of these intracellular domains[38,39].

All of the cytokines that utilize the IL2R common γ chain (γ_c_), have been found to be critical both for their function in the periphery after maturation, and for lymphoid development as well. In particular, mutations of the IL7R α chain, or the γ_c _chain, or its associated kinase, JAK3, are the major causes of human severe combined immunodeficiency (SCID)[18]. Three signaling pathways are known to be activated via the γc chain, the Ras/Raf/MAPK pathway[40], the PI3K/Akt pathway[41], and the STAT5 pathway[42]. All evidence pointed to the STAT5a/b molecules as being the primary signalers of lymphocyte development as well as being critical for promoting cell cycle progression of mature lymphocytes. However, the importance of STAT5a/b in transmitting signals from IL7Rs to developing lymphocytes became controversial, primarily because mice that had a deletion of the N-terminal exon of the STAT5a/b molecules had relatively normal lymphocyte development[43]. However, these mice still expressed a truncated and partially functional STAT5 protein.

Now, utilizing mice that had total deletions of the entire STAT5a/b loci, John O'Shea's group[38] and Veronika Sexl's group[39] have both reported that the phenotype of these mice is as expected, severe combined immunodeficiency. In addition, Sexl's group reports that these mice are not susceptible to malignant transformation by the Src family kinase, Abelson. This last finding is extremely important, for it provides the missing link between the Src family kinases and transformation that has eluded investigators for over 25 years. In addition, these data are consistent with the known functions of STAT5, to activate the program for cell cycle progression through G_1 _to S-phase, primarily by promoting the expression and activity of the G_1 _D_2 & 3_-type cyclins[42]. In this regard, it is noteworthy that v-src, which phosphorylates STAT3 constitutively, has recently been found capable of transforming cells by activating the expression of cyclin D_1_[44]. It is now predictable that the identification of STAT inhibitors may well be very effective anti-cancer agents, and perhaps immunosuppressive agents as well.
